# Distribution of Dimethylsulfoniopropionate Degradation Genes Reflects Strong Water Current Dependencies in the Sanriku Coastal Region in Japan: From Mesocosm to Field Study

**DOI:** 10.3389/fmicb.2020.01372

**Published:** 2020-07-13

**Authors:** Yingshun Cui, Shu-Kuan Wong, Ryo Kaneko, Ayako Mouri, Yuya Tada, Ippei Nagao, Seong-Jun Chun, Hyung-Gwan Lee, Chi-Yong Ahn, Hee-Mock Oh, Yuki Sato-Takabe, Koji Suzuki, Hideki Fukuda, Toshi Nagata, Kazuhiro Kogure, Koji Hamasaki

**Affiliations:** ^1^Marine Microbiology, Department of Marine Ecosystem Dynamics, Atmosphere and Ocean Research Institute, The University of Tokyo, Kashiwa, Japan; ^2^National Institute of Polar Research, Tachikawa, Japan; ^3^National Institute for Minamata Disease, Kumamoto, Japan; ^4^Graduate School of Environmental Studies, Nagoya University, Nagoya, Japan; ^5^Cell Factory Research Center, Korea Research Institute of Bioscience and Biotechnology (KRIBB), Daejeon, South Korea; ^6^National Institute of Ecology, Seocheon-gun, South Korea; ^7^Faculty of Environmental Earth Science, Hokkaido University, Sapporo, Japan; ^8^Coastal Conservation, International Coastal Research Center, Atmosphere and Ocean Research Institute, The University of Tokyo, Tokyo, Japan; ^9^Marine Biogeochemistry, Department of Chemical Oceanography, Atmosphere and Ocean Research Institute, The University of Tokyo, Kashiwa, Japan; ^10^Collaborative Research Institute for Innovative Microbiology, The University of Tokyo, Tokyo, Japan

**Keywords:** dimethyl sulfide, dimethylsulfoniopropionate, *dddD* gene, *dddP* gene, *dmdA* gene, mesocosm, Sanriku Coast

## Abstract

Dimethyl sulfide (DMS) is an important component of the global sulfur cycle as it is the most abundant sulfur compound that is emitted *via* the ocean surface to the atmosphere. Dimethylsulfoniopropionate (DMSP), the precursor of DMS, is mainly produced by phytoplankton and is degraded by marine bacteria. To reveal the role of bacteria in the regulation of DMSP degradation and DMS production, mesocosm and field studies were performed in the Sanriku Coast on the Pacific Ocean in northeast Japan. The responsible bacteria for the transformation of DMSP to DMS and the assimilation of DMSP were monitored, and the genes encoding DMSP lyase (*dddD* and *dddP*) and DMSP demethylase (*dmdA*) were analyzed. The mesocosm study showed that the *dmdA* subclade D was the dominant DMSP degradation gene in the free-living (FL) and particle-associated (PA) fractions. The *dddD* gene was found in higher abundance than the *dddP* gene in all the tested samples. Most importantly, DMS concentration was positively correlated with the abundance of the *dddD* gene. These results indicated that bacteria possessing *dmdA* and *dddD* genes were the major contributors to the DMSP degradation and DMS production, respectively. The genes *dmdA* subclade D and *dddP* were abundant in the Tsugaru Warm (TW) Current, while the *dmdA* subclade C/2 and *dddD* genes were dominant in the Oyashio (OY) Current. Functional gene network analysis also showed that the DMSP degradation genes were divided into OY and TW Current-related modules, and genes sharing similar functions were clustered in the same module. Our data suggest that environmental fluctuations resulted in habitat filtering and niche partitioning of bacteria possessing DMSP degradation genes. Overall, our findings provide novel insights into the distribution and abundance of DMSP degradation genes in a coastal region with different water current systems.

## Introduction

**FIGURE 1:**
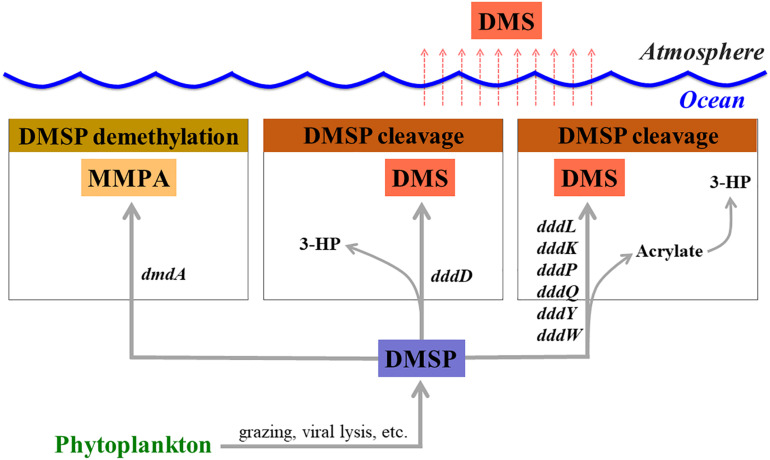
Genes involved in dimethylsulfoniopropionate (DMSP) degradation and dimethyl sulfide (DMS) production. MMPA, methylmercaptopropionate; 3-HP, 3-hydroxypropionate.

Dimethylsulfoniopropionate (DMSP) is mainly produced by marine phytoplankton and macroalgae as an osmolyte (Pichereau et al., 1998) and is an essential sulfur source for microbes in the seawater (Kiene et al., 1999). Once it is released into seawater, dissolved DMSP (DMSPd) mainly undergoes two different metabolic pathways mediated by bacteria (demethylation and cleavage pathways) (Curson et al., 2011; Moran et al., 2012; Figure 1). Marine phytoplankton and other organisms also degrade a small portion of DMSP (Stefels, 2000). It has been reported that over half of the marine bacteria groups, including SAR11, SAR116, *Gammaproteobacteria*, and *Roseobacter* actively degrade DMSP *via* the demethylation pathway using a DMSP demethylase encoded by the *dmdA* gene (Howard et al., 2006, 2008, 2011; Levine et al., 2012; Varaljay et al., 2012). In the DMSP cleavage pathway, dimethyl sulfide (DMS) is produced as a final metabolite and released back into the seawater (Curson et al., 2011; Moran et al., 2012). Upon release, a large portion of DMS will be utilized or catabolized by marine bacteria (Boden et al., 2010; Schafer et al., 2010), while the remaining DMS is released into the atmosphere (Lovelock et al., 1972). This sea-to-air DMS flux represents about 50% of the global biogenic sulfur flux (Andreae, 1990). In this way, bacterial activities related to DMSP degradation and DMS production can be directly linked to the biogenic sulfur cycles in the oceans. Therefore, it is essential to identify the factors that regulate the activity of DMSP-degrading bacteria in order to fully understand the global sulfur cycles.

Previous studies have revealed that bacterial DMSP metabolism is strongly influenced by environmental factors. Levine et al. (2012) reported that bacterial DMSP degradation and DMS production significantly increased with elevation of temperature and low dose of UV-A in the Sargasso Sea. In a different study performed at the Station ALOHA, it was shown that water depth-related parameters, including DMS, particulate DMSP (DMSPp), chlorophyll *a* (Chl *a*), and dissolved organic carbon concentrations together with the DMSP-to-Chl *a* ratio, could be the essential factors that are positively related to the abundance of bacteria harboring *dmdA* and *dddP* genes (Varaljay et al., 2012). Our previous work identified spatial variabilities in the distribution of bacteria possessing *dmdA* and *dddP* genes in the low-latitude region of the Pacific Ocean and possible correlation of their abundance with Chl *a* concentration and negative correlation with temperature (Cui et al., 2015). However, the bacterial communities differ largely among oceanic regions such as between the subtropical and subarctic regions in the North Pacific Ocean (Taniguchi and Hamasaki, 2008). They found that Roseobacter-related and Cytophaga-Flavobacterium-Bacteroides (CFB) group phylotypes were the major actively growing bacteria in both subarctic and subtropical western North Pacific, and *Gammaproteobacteria* and Gram-positive bacteria were the major groups in subtropical stations. Additionally, phytoplankton blooming conditions could also alter the bacterial community compositions in the western North Pacific (Tada et al., 2011). However, the exact factors that control the distribution and dynamics of DMSP-degrading bacteria in subarctic regions of the Pacific Ocean are still unknown.

The Sanriku Coast, facing the western North Pacific, is influenced by spatiotemporal fluctuations of two major current systems. The southward-flowing Oyashio (OY) Current carries cold, low-salinity, and nutrient-rich waters to the Sanriku coastal area, while the Tsugaru Warm (TW) and Kuroshio Currents carry saline and warm subtropical waters (Hanawa and Mitsudera, 1986; Wagawa et al., 2015). These dynamic water current systems form complex hydrographic structures and are believed to be responsible for the diverse and highly productive ecosystems in the Sanriku Coast. Previous studies have revealed that the composition of the bacterial communities, bacterial activities, and their potential metabolism are strongly influenced by the water currents in the subarctic western Pacific Ocean (Tada et al., 2011; Li et al., 2018). These findings indicate that water currents influence both phytoplankton growth and nutrient concentrations, which could shape the bacterial community structures. Therefore, the Sanriku Coast, characterized by two major current systems with clear seasonality, could be an ideal place to investigate the factors that regulate bacterial DMSP metabolism in relation to environmental fluctuation.

In this study, genes encoding the DMSP lyase (*dddD* and *dddP*) and DMSP demethylase (*dmdA*) were analyzed, and the major DMS producers were probed. We collected field samples in different seasons to identify the dynamics of DMSP metabolism and DMS production in relation to different water masses. We also performed mesocosm experiments using water samples from the Sanriku Coast to investigate the dynamics of DMSP-degrading bacteria in response to phytoplankton blooms.

## Materials and Methods

### Mesocosm Setup, Sampling, and DNA Extraction

Since DMSP production varies among different phytoplankton taxa (Matrai and Keller, 1994; Niki et al., 2000), we designed two different mesocosm conditions: Mesocosm 1 was aimed to induce coccolithophore, and Mesocosm 2 aimed to induce diatom blooms. Seawater from 42 m depth, corresponding to the subsurface Chl *a* maxima, was collected from the Otsuchi Bay (39° 21′ N, 141° 57′ E, Western North Pacific Ocean, Japan), near the station OT3, on June 7, 2010 (Supplementary Figure S1). Four mesocosm 200-L cylindrical polycarbonate tanks were divided into the two groups, Mesocosm 1 and Mesocosm 2, according to the conditions listed hereafter. Tanks A and B, designated as Mesocosm 1, contained seawater filtered through a 10 μm pore size filter to remove large phytoplankton. On top of the original organic nutrients already present in the filtered seawater, additional inorganic nutrients (NaNO3 and NaH2PO4 with final concentrations of 44.1 and 4.4 μM, respectively) were added into this mesocosm. In addition, an axenic strain of *Gephyrocapsa oceanica* NIES-353, the most abundant coccolith species in marginal seas and the neritic zone (Okada and Honjo, 1975; Tanaka, 1991; Bollmann, 1997), was pre-cultured in laboratory conditions and added in Mesocosm 1 as a seed population. The final concentration of Chl *a* was adjusted to approximately 1.7 μg L^–1^. Seawater in the tank was slowly agitated with a polyvinyl chloride rod before each sampling. Tanks C and D were designated as Mesocosm 2 and contained seawater that was sieved through a 100 μm nylon mesh to remove mesozooplankton. Sodium silicate (Na_2_SiO_3_, final concentration was adjusted to 2.64 μM) was added in Mesocosm 2 to accelerate the growth of native diatoms. All tanks were kept in an outdoor reservoir of running seawater pumped up from the shore and incubated for 10 days at *in situ* seawater temperature under natural daylight.

Approximately 1 L of seawater samples were collected from each tank at Days 0, 2, 4, 5, 6, 8, 9, and 10 for DNA extraction. Upon collection, the samples were immediately filtered through 47 mm diameter, 3.0 μm pore size Nuclepore^TM^ filters (Whatman^®^, Clifton, NJ, United States) and sequentially filtered through 0.22 μm pore size Sterivex^TM^ filter units (Millipore^TM^, Bedford, MA, United States). We designated the size fractions between 0.22 and 3 μm as the free-living (FL) fractions, and the > 3 μm fractions as the particle-associated (PA) ones. The filters were stored at −20°C until further analysis. DNA was extracted using a ChargeSwitch^®^ Forensic DNA Purification Kit (Invitrogen, Carlsbad, CA, United States) according to the manufacturer’s instructions.

To analyze phytoplankton pigments, 1-L seawater samples were collected from each tank at Day 7 and filtered through Whatman GF/F filters (25 mm in diameter) (Whatman^®^, Clifton, NJ, United States) under a gentle vacuum (<0.013 MPa). The filters were stored at −80°C until further analysis.

### Biological and Physicochemical Factors

Concentrations of DMS and DMSP were measured by gas chromatography following the method described previously (Simo et al., 1996; Nagao et al., 2009). For DMS measurement, 30 ml seawater samples collected from each tank were introduced into a degasification vessel by syringe through a filter (DISMIC PTFE, ADVANTEC, Japan) and purged by N_2_ gas. The extracted gas underwent a series of processes (ca. removing of water vapor and concentration) and was detected using gas chromatography equipped with a flame photometric detector (GC-14B, Shimadzu Co. Ltd., Japan). For the measurement of DMS plus total DMSP (DMSPt), seawater samples of 15–33 ml were collected directly into glass vials and were subjected to an alkali treatment using sodium hydroxide at pH 12 for 6 h to permit cleavage into gaseous DMS. The gaseous DMS was measured in the same manner as mentioned above. To determine the DMSPp concentration, seawater samples were filtered gravitationally through GF/F filters (Whatman^®^, Clifton, NJ, United States). The filters were then immersed in sealed brown glass vials containing sodium hydroxide solution (pH 12) and kept under room temperature for 6 h to permit the cleavage of DMSPp into gaseous DMS. The gaseous DMS was measured as described above. The concentration of the DMSPd was calculated by subtracting the DMSPp concentration from the DMSPt concentration.

To determine the Chl *a* concentration, duplicate seawater samples were filtered using Whatman GF/F filters (Whatman^®^, Clifton, NJ, United States). Chl *a* was extracted with *N*,*N*-dimethylformamide for 24 h in the dark at 4°C, and its concentration was determined fluorometrically (Holm-Hansen et al., 1965; Suzuki and Ishimaru, 1990; Tada et al., 2012). Total bacterial abundance in the FL and PA fractions was determined with the 4′,6′-diamidino-2-phenylindole (DAPI) staining method (Suzuki et al., 1993) (final concentration: 2 μg ml^–1^) in the dark and counted (Porter and Feig, 1980) using an epifluorescence microscope (Olympus BX-51; Olympus Optical, Tokyo, Japan). A total of 20 ml of paraformaldehyde-fixed liquid samples were filtered through 3.0 μm pore size Nuclepore^TM^ filters (Whatman^®^, Clifton, NJ, United States) to collect the bacteria in the PA fractions. From this filtrate, 5–10 ml were subsequently filtered through 0.22 μm pore-size polycarbonate black membrane filters (25 mm, type GTBP; Millipore, Cork, Ireland) to collect the bacteria in the FL fractions. All filters were stored at −20°C until further processing.

Analysis of phytoplankton pigments was performed using high-performance liquid chromatography (HPLC) following the method previously described (Endo et al., 2013). To estimate the contribution of selected algal classes to the phytoplankton in each experiment on Day 7, a multiple linear regression analysis was performed using the concentrations of Chl *a* and algal chemotaxonomic pigments for each group (Barlow et al., 1993; Suzuki et al., 1997). Chl *a* was used as an indicator of total phytoplankton biomass. In this study, we assumed that the major chemotaxonomic pigments fucoxanthin (Fuco), 19′-hexanoyloxyfucoxanthin (19′-Hex), and Chl *b* derived mainly from diatoms, haptophytes, and green algae (prasinophytes and chlorophytes), respectively (Jeffrey, 1974; Wright and Jeffrey, 1987; Liu et al., 2009). The regression equation was set as follows:

$$\left[\mathrm{Chl}\,a\right]=a\,\left[\mathrm{Fuco}\right]+b\,\left[19^{\prime }\,\text{-Hex}\right]+c\,\left[\mathrm{Chl}\,b\right]+d$$

The dimensionless coefficients of *a*, *b*, *c*, and the constant term *d* were determined by the LINEST function of Microsoft Excel. *F*-test and *t*-statistics were also carried out to validate the regression equation obtained and to confirm whether or not each coefficient was reliable to the regression analysis, respectively.

### Design of Primer Set Targeting *dddD* and Clone Library Construction

The *dddD* sequence reported from *Marinomonas* sp. MWYL1 was used as the query sequence for BLASTX analysis. A total of 29 different sequences with >40% of amino acid identities obtained from BLASTX were then aligned using CLUSTAL X, and the conserved regions were used to design the *dddD* gene primer sets for this study. The sequences of the degenerate primer set used in this study were as follows: *dddD*_1360F: 5′-AACGTBATHGCHGGBCCBCAYTC-3′ and *dddD*_1799R: 5′-GTVCCGARRTGVGCGTGYTCYTC-3′. The *dddD* primers’ specificity was confirmed by testing the designed primers against several *dddD*-possessing bacterial isolates (*Hoeflea phototrophica* DSMZ17068, *Marinomonas mediterranea* MMB-1, *Marinomonas* sp. MWYL1, *Oceanimonas doudoroffii* JCM21046, *Pseudomonas* sp. J465, *Psychrobacter* sp. J466, *Sinorhizobium fredii* NGR234, *Burkholderia phymatum* STM815). PCR using the newly designed primer set was also performed using *Sulfitobacter* sp. EE-36 and *Roseovarius nubinhibens* ISM as the negative control. Since *dddD* gene was absent from these strains, no PCR amplification for *dddD* gene was detected for these strains using the newly designed primer set. In addition, field samples collected from the open ocean (Pacific Ocean) were used as negative control since it was known that the *dddD* gene abundance was extremely low in the open ocean.

To construct the *dddD* clone libraries from our mesocosm seawater samples, PCR products were amplified with the above primer set, ligated, and inserted into a TOPO-TA cloning vector (Invitrogen) according to the manufacturer’s instructions. The colonies were then amplified with the M13 primer set and subjected to Sanger sequencing. PCR artifacts/chimeras were removed by using pre.cluster and chimera.uchime steps in the “mothur v. 1.39.3” software prior to alignment. Phylogenetic relationships among reference and newly obtained *dddD* sequences were inferred from multiple alignments by using CLUSTAL X with the default settings. Phylogenetic trees were calculated using the neighbor-joining method with the MEGA 5.1 software (Tamura et al., 2011). Sequences with > 90% similarity were designated as a single cluster.

### Quantification of Dimethylsulfoniopropionate Degradation Genes

The copy numbers of DMSP degradation genes from mesocosm seawater samples were analyzed by qPCR using the Bio-Rad Chromo4^TM^ System. Eight *dmdA* primer sets were designed to target the different subclades, A/1, A/2, B/3, B/4, C/2, D/1, D/3, and E/2 (Varaljay et al., 2010). Also a *dddP* primer set targeting the *Roseobacter* clade (Levine et al., 2012) and the newly designed *dddD* primer set were used to detect DMSP degradation genes. Prior to quantification, two DNA samples from each size fraction were subjected to sequencing in order to confirm the specificity of each primer set.

The qPCR assay for *dddD*, *dddP*, and *dmdA* subclades was performed in 20 μl mixtures containing 10 μl of 2× SYBR Premix Ex Taq (Tli RNaseH Plus) kit (TaKaRa Bio Inc., Otsu, Japan), 0.4 μl of 50× ROX reference dye, 0.2 mM of each primer (for *dddD*, 0.6 mM of each primer), 2 μl of 1/10-diluted DNA template (ranges of FL template were 285–7,388 ng ml^–1^ and PA 117–847 ng ml^–1^ in final concentration), and 6.8 μl of double distilled water. The qPCR conditions have been previously described (Cui et al., 2015). For *dddD*, the annealing temperature was set at 61°C. The concentrations of the qPCR standards were determined using the Quant-iT^TM^ PicoGreen^®^ dsDNA Kit (Invitrogen). A 10-fold serially diluted standard (range: 10^0^–10^7^ copy numbers ml^–1^ of each standard) and a no-template control were tested in triplicate for each reaction. The presence of a single band was verified by agarose gel electrophoresis.

### Field Study

Water samples were collected from January 2013 to April 2014 in two to three different sites (OT3, OT4, and OT5) (Table 1 and Supplementary Figure S1) of the Sanriku Coast during the cruises of R/V Shinsei Maru. Temperature, salinity, and depth were measured onboard using conductivity-temperature-depth (CTD) sensors. Chl *a* concentration was determined as described above. Water samples for nutrient analysis were collected in acid-cleaned polypropylene tubes and stored at −20°C for further analysis. Ammonia, nitrate, nitrite, phosphate, and silicate concentrations were determined by an autoanalyzer (AACS II or AACS III; Bran+Luebbe, Germany) as previously described (Fukuda et al., 2016). Particulate organic carbon (POC) and nitrogen (PON) were determined as previously described (Miyajima et al., 1998). Seawater for DNA analyses was collected from the surface (0 m) of the sea and 50 m depth. Filtration of seawater onboard for DNA samples, DNA extraction procedures, and the quantification of DMSP degradation genes were performed as mentioned above. In order to isolate *dddD* sequences from the field samples, PCR experiments were performed where the gene was amplified for 20 cycles using the following bar coded primer set *dddD*_1360F: 5′-CCATCTCAT CCCTGCGTGTCTCCGACTCAGxxxxAACGTBATHGCHGGB CCBCAYTC-3′ and *dddD*_1799R: 5′-GTVCCGARRTGVGCG TGYTCYTC-3′; where *x*s represent the bar code sequences. Adapter sequences are double underlined, while primer sequences are underlined. PCR products were prepared in triplicates and pooled before purification. After the purification and quantification of extracted DNA, 454 pyrosequencing was performed following the procedure described previously (Cui et al., 2015). The obtained sequence data were processed using the “mothur v. 1.39.3” software, including primer and bar code searches and alignment against a newly created *dddD* database (see Supplementary Methods). Obtained *dddD* sequences and accompanying metadata have been deposited in the Sequence Read Archive (SRA) of NCBI under project number PRJNA547684.

**TABLE 1:** Sampling sites, depth, and biophysicochemical parameters of the field study.

Year	Month	Sample ID	Longitude (N)	Latitude (E)	Depth	Salinity	Temperature	Chl *a*	Nitrate	Nitrite	Ammonia	Phosphate	Silicate	POC	PON
					(m)	(psu)	(°C)	(μg l^–1^)	(μM)	(μM)	(μM)	(μM)	(μM)	(μM)	(μM)
2013	JAN	Jan-OT3-0 m	141.98	39.36	0	33.86	9.83	0.34	5.88	0.07	0.2	0.48	9.1	3.44	0.31
		Jan-OT3-50 m	141.98	39.36	50	33.81	9.47	0.64	5.53	0.14	0.2	0.47	9.1	4.88	0.6
		Jan-OT5-0 m	142.5	39.33	0	33.84	9.3	0.43	6.47	0.06	0.2	0.52	10.2	2.98	0.38
		Jan-OT5-50 m	142.5	39.33	50	33.83	9.24	0.41	6.63	0.06	0.1	0.54	10.7	4.43	0.66
	JUN	Jun-OT3-0 m	141.98	39.36	0	33.57	14.83	0.77	0	0	0	0	0	11.3	1.65
		Jun-OT3-50 m	141.98	39.36	50	33.76	13.4	1.34	0	0	0	0	0	11.8	1.58
		Jun-OT5-0 m	142.5	39.33	0	33.67	16.37	0.51	0	0	0.1	0.02	1.4	9.06	1.29
		Jun-OT5-50 m	142.5	39.33	50	33.87	10.29	0.98	2.74	0.36	0.22	0.27	4.7	4.94	0.83
	SEPT	Sept-OT3-0 m	141.98	39.36	0	33.06	22.38	0.61	0.54	0.05	0.1	0.03	4.9	8.43	1.5
		Sept-OT3-50 m	141.98	39.36	50	33.57	20.99	0.42	1.2	0.29	0.4	0.21	4.8	6.56	0.73
		Sept-OT4-0 m	142.5	39.33	0	33.23	19.13	0.82	0.16	0.03	0	0.12	3.1	7.08	1.05
		Sept-OT4-50 m	142.5	39.33	50	34.09	11.45	0.07	7.7	0	0	0.58	12.2	1.57	0.18
	DEC	Dec-OT3-0 m	141.98	39.36	0	33.52	13.63	0.62	3.52	0.59	0.1	0.29	7.3	NA	NA
		Dec-OT3-50 m	141.98	39.36	50	33.52	13.64	0.62	3.51	0.6	0.1	0.28	7.2	NA	NA
		Dec-OT4-0 m	142.17	39.33	0	33.56	13.88	0.22	4.03	0.23	0.1	0.28	7.3	NA	NA
		Dec-OT4-50 m	142.17	39.33	50	33.61	13.82	0.18	4.48	0.33	0.1	0.32	8.4	NA	NA
2014	MAR	Mar-OT3-0 m	141.98	39.36	0	32.77	3.58	0.87	11.36	0.2	0.3	0.96	22.9	6.87	1.34
		Mar-OT3-50 m	141.98	39.36	50	33.03	3.84	0.28	11.44	0.22	0.6	0.98	22.7	2.54	0.43
		Mar-OT4-0 m	142.17	39.33	0	32.75	2.08	5.58	11.76	0.16	0.2	1.09	25.6	11.2	2.07
		Mar-OT4-50 m	142.17	39.33	50	32.88	2.3	0.41	16.36	0.16	0.2	1.36	31.3	2.21	0.39
		Mar-OT5-0 m	142.5	39.33	0	32.82	1.47	4.09	15.13	0.17	0.1	1.35	31.7	13.2	2.4
		Mar-OT5-50 m	142.5	39.33	50	33.12	2.52	0.22	19.38	0.13	0.1	1.59	37.1	2.12	0.35
	APR	Apr-OT3-0 m	141.98	39.36	0	32.56	4.44	5.47	3.65	0.07	0.9	0.53	5	NA	NA
		Apr-OT3-50 m	141.98	39.36	50	32.59	4.07	9.94	0.34	0.03	0.1	0.21	1.5	NA	NA
		Apr-OT4-0 m	142.17	39.33	0	32.4	2.34	1.49	20.28	0.2	0.3	1.68	36.9	NA	NA
		Apr-OT4-50 m	142.17	39.33	50	32.89	1.21	31.2	0.35	0.03	0.1	0.42	5	NA	NA
		Apr-OT5-0 m	142.5	39.33	0	32.48	2.33	1.38	18.3	0.15	0.5	1.55	33.2	NA	NA
		Apr-OT5-50 m	142.5	39.33	50	32.81	1.22	42.7	0.99	0.03	0.1	0.47	7.3	NA	NA

*Chl a, chlorophyll a; NA, not available; POC, particulate organic carbon; PON, particulate organic nitrogen.*

### Statistical Analysis

To assess the succession patterns of *dddD* genes and DMSP degradation genes in FL and PA fractions, nonmetric multidimensional scaling (NMDS) was performed using the “Vegan” package implemented in the R software version 3.4.4 (Oksanen et al., 2010). To perform NMDS, Bray–Curtis dissimilarities were calculated using the abundance of each *dddD* gene cluster (in the mesocosm study) after arcsine square root transformation and DMSP degradation genes (in the field study) after logarithmic transformation. Then, to determine the environmental drivers of the distribution of DMSP degradation genes, environmental factors were fitted to the NMDS ordination in the field study. The *envfit* functions in the Vegan package were used to evaluate the fit (*R*^2^) of environmental factors to the NMDS ordination (999 permutations).

To examine the relationship among the multiple DMSP degradation genes and environmental factors, an associated network was constructed based on Spearman’s correlation. The correlation coefficient (*ρ*) and significance values (*P*) were calculated using the R software (package: Hmisc) (Harrell et al., 2007). Only the correlations with *P* < 0.001 and |*ρ*| ≥ 0.6 were retained for the construction of the association network. Erdös–Rényi random networks, which had the same number of nodes and edges as the real co-occurrence networks, were generated with each edge having the same probability of being assigned to any node (Erdõs and Rényi, 1960), and the average value of each network metric was reported. Topological properties of both random and real networks, including clustering coefficient, averaged shortest path length, averaged node degree, network density, diameter, and modularity (modules were identified by using the Louvain algorithm), were calculated with the R package “igraph” (Csardi and Nepusz, 2006). The network was visualized using Cytoscape 3.5.1 (Shannon et al., 2003).

## Results

Mesocosm experiments using two different phytoplankton blooms, coccolithophore, a group of calcifying unicellular microalgae often dominating phytoplankton biomass in subarctic open ocean (Balch, 2018), and diatom, a major phytoplankton in the ocean, were performed in order to (1) investigate the variability of DMSP degradation and DMS emission patterns in relation to the different blooms and (2) explore the dynamics of the bacterial community compositions and DMSP-degrading bacteria in different phytoplankton blooms and their derived biochemical factors. In addition, field samples were collected in different seasons (1) to identify the major DMSP degraders and DMS producers in relation to different water masses of the Sanriku Coast, (2) to explore the drivers of distribution and abundance of DMSP degradation genes, and (3) to uncover functional gene networks among different DMSP degradation genes.

### Mesocosm Study

#### Biophysicochemical Factors in Two Mesocosm Systems

**FIGURE 2:**
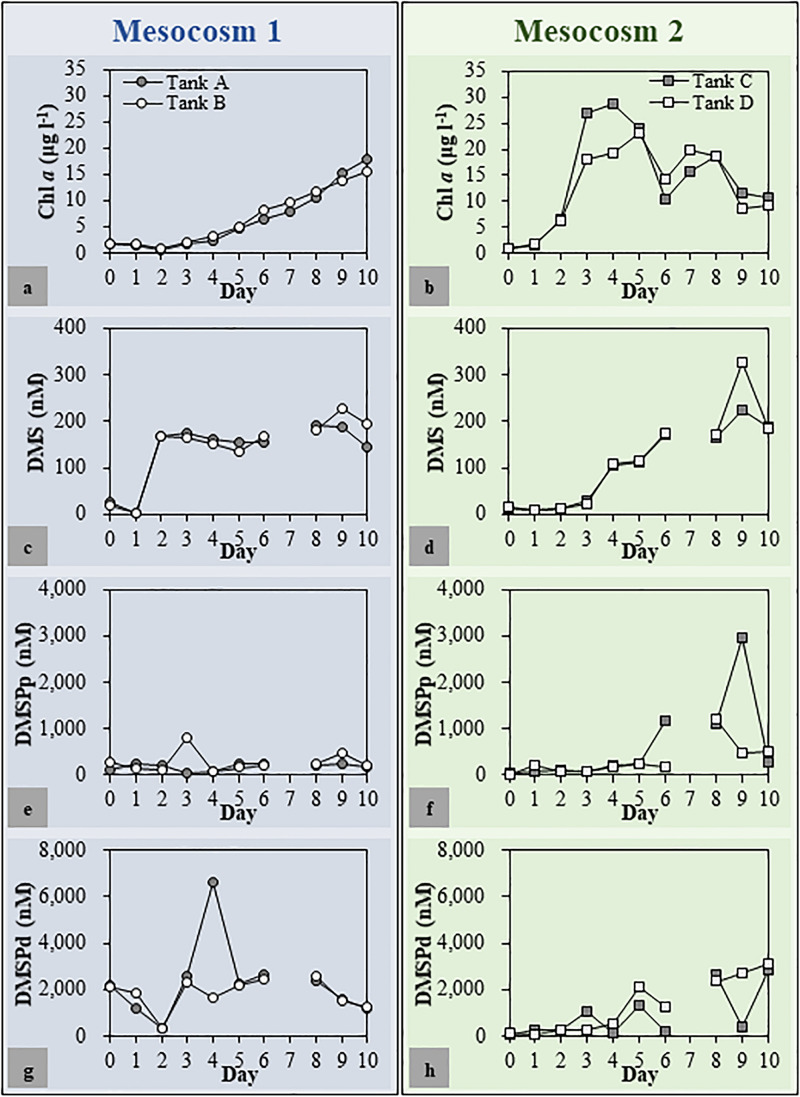
Chlorophyll *a* (Chl *a*) **(a,b)**, dimethyl sulfide (DMS) **(c,d)**, dimethylsulfoniopropionate (DMSP) [particulate DMSP (DMSPp) **(e,f)** and dissolved DMSP (DMSPd) **(g,h)**] concentrations in each mesocosm tank. The patterns of change in environmental parameters throughout the mesocosm period were similar for duplicated mesocosm groups. Mesocosm 1, Tanks **(a,b)**; Mesocosm 2, Tanks **(c,d)**.

The Chl *a*, DMSPp, DMSPd, and DMS concentrations as well as the bacterial abundance showed resemblance between the duplicated mesocosm tanks, while these factors varied in the different mesocosm groups (Figure 2). In Mesocosm 1, the Chl *a* concentration increased steadily until the end of the mesocosm experiment (from 1.75 ± 0.03 to 16.68 ± 1.26 μg l^–1^). In Mesocosm 2, a typical growth-and-collapse of phytoplankton blooms was observed after a Chl *a* peak of approximately 24.07 ± 4.81 μg l^–1^. The initial DMSPp and DMSPd concentrations were approximately 6 and 21 times higher, respectively, in Mesocosm 1 than in Mesocosm 2. This variation was most likely caused by the initial addition of coccolithophores as seed phytoplankton in Mesocosm 1. Concentrations of DMSPp and DMSPd mostly matched between duplicate tanks but varied at some time points in both mesocosm systems. Unusually high concentrations of DMSPd at Day 3 in Mesocosm 1 (6,617 nM) and DMSPp at Day 8 in Mesocosm 2 (2,722 nM) were observed. The high concentration of DMSPd could be related to the phytoplankton lysis that caused the sudden release of DMSPd. DMS concentrations increased steadily during the experiment in Mesocosm 2, with the highest concentration of 275 ± 50 nM observed at Day 9. On the other hand, DMS concentrations in Mesocosm 1 sharply increased from Days 1 to 2, and high concentrations were maintained for several days. Then, the concentration increased again and peaked at Day 9 (208 ± 19 nM).

After 7 days of incubation, fucoxanthin, an accessory pigment used as a marker for the presence of diatoms (Wright and Jeffrey, 1987), was the most abundant pigment in both Mesocosms 1 and 2 (Supplementary Table S1) followed by 19′-hexanoyloxyfucoxanthin, an accessory photosynthetic pigment found exclusively in haptophytes (Liu et al., 2009), in Mesocosm 1 and Chl *b*, an accessory pigment in green algae such as prasinophytes and chlorophytes (Jeffrey, 1974), in Mesocosm 2. Notably, the concentration of 19′-hexanoyloxyfucoxanthin was much higher in Mesocosm 1 than that in Mesocosm 2.

An initial adaptation period of FL bacterial populations to a new environment was observed in the mesocosm experiments (Supplementary Figure S2). Bacterial abundance in the FL fractions decreased until Day 4, then increased rapidly, and peaked at Day 7. Bacterial abundance in the PA fractions was higher in Mesocosm 2 compared to that in Mesocosm 1. From Day 6 onward, the formation of large aggregates made the process of counting bacterial cells in the PA fractions almost impossible.

#### Dimethylsulfoniopropionate Lyase (*dddD* and *dddP*) Gene Abundance and Dimethyl Sulfide Production

**FIGURE 3:**
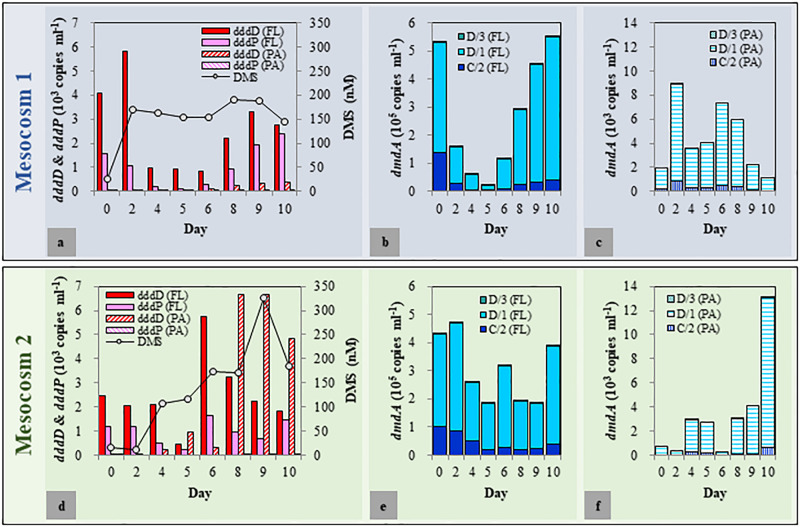
Abundance of dimethylsulfoniopropionate (DMSP) degradation genes in free-living (FL) and particle-associated (PA) fractions and changes in the dimethyl sulfide (DMS) concentrations during the mesocosm experiment. **(a–c)** Mesocosm 1; **(d–f)**: Mesocosm 2.

As biological and physicochemical factors in the duplicated mesocosm tanks revealed high resemblance within each group, one of each mesocosm group (Tank A and Tank D as representatives for Mesocosm 1 and Mesocosm 2, respectively) was chosen to quantify the DMSP degradation genes and to explore the variations of *dddD* genes (to be described later). The *dddD* genes were up to eight times more abundant than those of *dddP* in both mesocosm systems (Figures 3a,d). These two genes were approximately one to two orders of magnitude more abundant in the FL fractions than in the PA fractions except for a few samples from the latter phase of the experiment in Mesocosm 2. In the FL fractions of Mesocosm 1, the initial abundance of *dddD* (4.09 × 10^3^ copies ml^–1^) increased until Day 2 (5.81 × 10^3^ copies ml^–1^) before decreasing until Day 6, while *dddP* genes (1.55 × 10^3^ copies ml^–1^) gradually decreased from Day 1 until Day 6. The abundances increased again during the latter phase of the experiment and reached 2.78 × 10^3^ and 2.38 × 10^3^ copies ml^–1^, respectively, at the end of the mesocosm experiment. In the PA fractions, *dddD* and *dddP* gene abundances were much lower (ranging from 33 to 531 copies ml^–1^) than those in the FL fractions. In Mesocosm 2, maximum *dddD* and *dddP* abundance was observed at Day 6 (5.73 × 10^3^ and 1.63 × 10^3^ copies ml^–1^, respectively) in the FL fractions, which decreased rapidly in the latter phase of the experiment. The decrease in the *dddD* gene abundance in the FL fractions was accompanied by a rapid increase in its abundance in the PA fractions from Day 8 (maximum of 6.68 × 10^3^ copies ml^–1^).

Most importantly, the changes in the concentration of DMS correlated well with the changes of the *dddD* gene abundances. More specifically, after initial decoupling, DMS concentrations correlated with total *dddD* gene abundance (sum of FL and PA fractions) (*r* = 0.56, *P* < 0.05), while *dddP* gene abundance did not (*r* = 0.04, *P* > 0.5).

#### Variations of the *dddD* Gene in the Different Size Fractions

**FIGURE 4:**
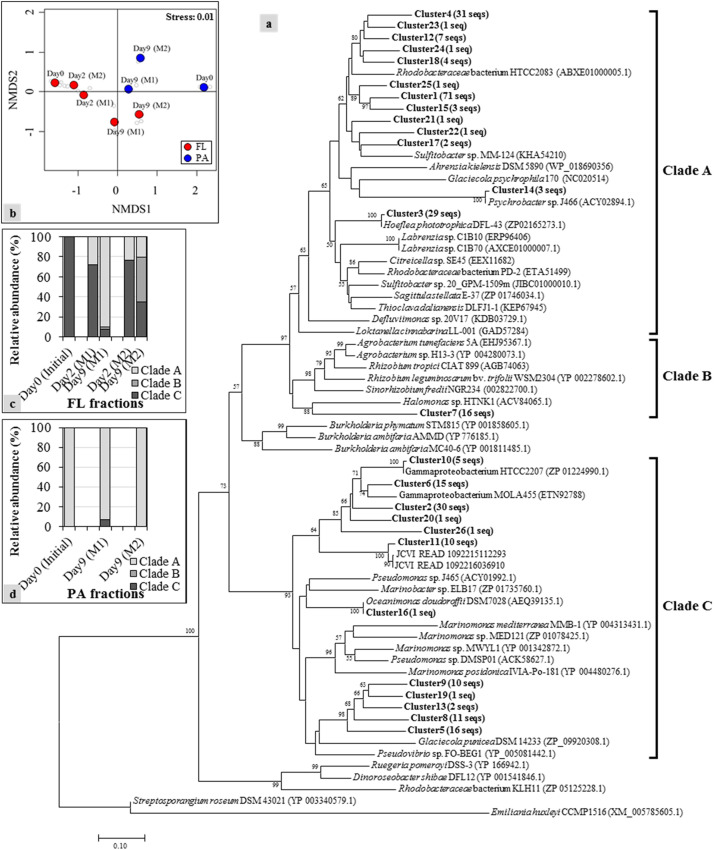
Neighbor-joining phylogenetic tree of *dddD* gene clusters (indicated as bold) with reference sequences obtained from the NCBI gene bank: **(a)** Clones with ≥ 90% sequence similarity were assigned to the same cluster. Numbers in parentheses next to each cluster indicate the number of sequences in that cluster. Bootstrap values > 50%, expressed as percentages of 1,000 replications, are shown at branch points. The phylogenetic tree is separated into several clades, including Clades A to C that contain sequences obtained from the current mesocosm study; **(b)** nonmetric multidimensional scaling (NMDS) constructed with the relative abundance of each *dddD* cluster showing the shifts of the *dddD* gene clades in free-living (FL) **(c)** and particle-associated (PA) **(d)** fractions from the different mesocosm tanks.

Based on the results obtained above, we assumed that the bacteria possessing *dddD* genes are the major DMS producers in both mesocosm systems. To explore the variations of *dddD* genes in different mesocosm systems and in different size fractions, *dddD* gene clone libraries were constructed with the samples from Day 0 (initial seawater), Day 2 (maximum DMS concentration observed in Mesocosm 1), and Day 9 (maximum DMS concentration observed in Mesocosm 2). Totally 267 *dddD* sequences were obtained and clustered into 26 different clusters using a 90% similarity threshold (Figure 4a). NMDS showed that the *dddD* clusters in the FL fractions significantly differed from those in the PA fractions (ANOSIM: *r* = 0.51, *P* = 0.016) (Figure 4b). The temporal succession patterns of *dddD* clusters varied more strikingly toward the end of the experiment in both mesocosm groups. A phylogenetic tree was constructed with potential *dddD* sequences that can be separated into several clades; Clade A or Rhodobacter clade was composed of bacteria assigned to the order of *Rhodobacterales*. Clade B or Rhizobium clade was composed of bacteria of the order of *Rhizobiales*, and Clade C or Gamma clade was composed of a wide variety of *Gammaproteobacteria*. All of the initial *dddD* genes belonged to Clade C in the FL fractions, and the abundance of genes from this clade decreased to 8 and 35% of the initial abundance of *dddD* genes at Day 9 in Mesocosm 1 and Mesocosm 2, respectively (Figure 4c). In contrast, genes from Clades A and B increased to 90 and 3% in Mesocosm 1 and 21 and 44% in Mesocosm 2, respectively. In PA fractions, Clade A dominated (>93%) in both mesocosm groups (Figure 4d).

#### Dimethylsulfoniopropionate Demethylase (*dmdA*) Gene Abundance in Two Mesocosm Systems

Among the seven subclades (A/1, A/2, B/3, C/2, D/1, D/3, and E/2) of *dmdA* genes, A/1, A/2, B/3, and E/2 were either under the detection limit or had less than 30 copies per ml (Figures 3b,c,e,f). The succession patterns of C/2, D/1, and D/3 differed between the two mesocosm systems as well as the FL and PA fractions. The abundance of these three subclades was approximately two orders of magnitude higher in the FL fractions than that in the PA fractions. In Mesocosm 1, the initial abundance of the *dmdA* subclades C/2, D/1, and D/3 decreased until Day 5. They were then recovered during the latter phase of the experiment. In Mesocosm 2, the abundance of these genes exhibited constant fluctuations in the FL fractions.

### Field Study

#### Water Properties of the Sanriku Coastal Area

**FIGURE 5:**
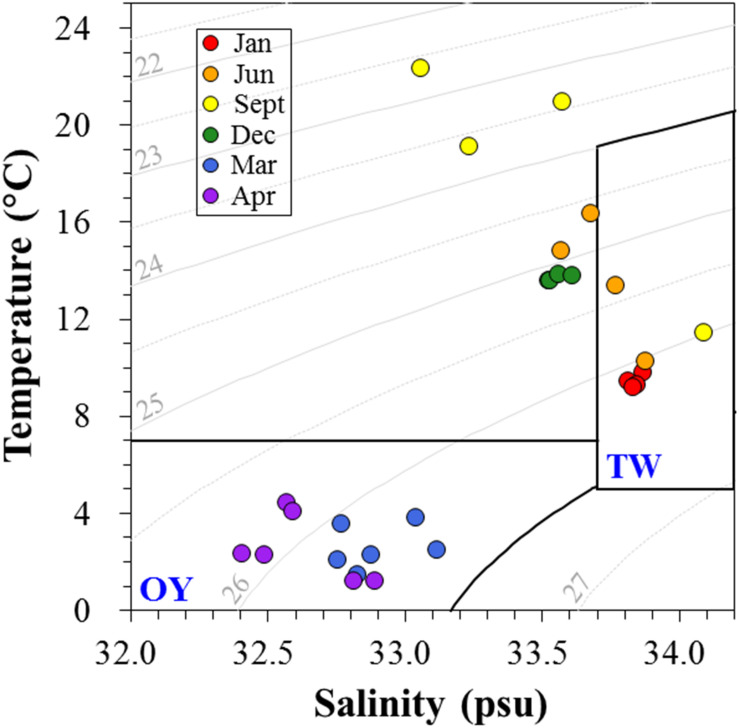
The temperature-salinity (TS) diagram depicts the presence of different water masses in the Sanriku coastal area during the sampling periods. The criteria proposed by Hanawa and Mitsudera (1986) were used to distinguish the typical water properties of the Oyashio (OY) and Tsugaru Warm (TW) Currents.

Environmental factors are summarized in Table 1. Temperature and salinity maxima were recorded in early fall, while the minima were recorded in spring. To distinguish the water mass depending on seasonal variations of the major current systems in the Sanriku coastal area, a temperature–salinity diagram (TS diagram) was constructed (Figure 5). The criteria established by Hanawa and Mitsudera (1986) were used to characterize the water mass. Based on this TS diagram, the spring samples (March and April) were within the range of the OY Current, which was characterized by low temperature (<7°C) and salinity (<33.7 psu). The samples collected in January, June, and September were within the range of the TW Current, which was characterized by relatively warm temperatures (>6°C) and high salinity (33.7–34.2 psu).

Chl *a* concentration was significantly higher in the surface waters in spring (ca. 4.1–31.2 μg l^–1^), while, at the samples taken during the other seasons, the Chl *a* concentrations were calculated to be less than 1.3 μg l^–1^. The highest nutrient concentration was also recorded in spring, while the lowest was observed in the surface waters in June (under detection limit). POC and PON also reflected the seasonal changes of water mass and were measured within the ranges of 1.6–13.2 μM and 0.2–2.4 μM, respectively. The nutrient concentrations at the surface of the waters were generally lower than those in 50 m depth. Several significant correlations were observed between different biophysicochemical factors. More specifically, nitrate, phosphate, and silicate concentrations were positively correlated with each other (*ρ* > 0.96, *P* < 0.001), and POC correlated with PON (*ρ* = 0.96, *P* < 0.001), while temperature was negatively correlated with salinity (*ρ* = 0.82, *P* < 0.001) with the exception of the values recorded in spring.

#### Distribution of Dimethylsulfoniopropionate Degradation Genes in Relation to Environmental Factors

**FIGURE 6:**
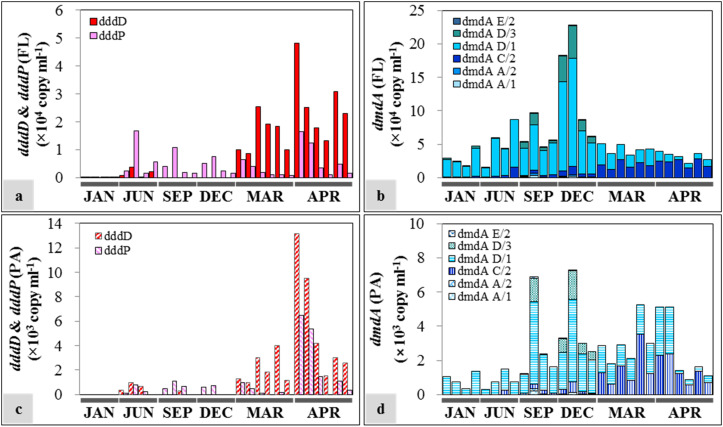
Seasonal distribution of dimethylsulfoniopropionate (DMSP) degradation genes in free-living (FL) and particle-associated (PA) fractions: **(a)** *dddD* and *dddP* abundances in the FL fractions; **(b)** *dmdA* abundance in the FL fractions; **(c)** *dddD* and *dddP* abundances in the PA fractions; **(d)** *dmdA* abundance in the PA fractions. The sample IDs are shown in Table 1. Each bar from left to right represents samples from the top to the bottom in Table 1.

The succession patterns of *dddD* and *dddP* highly reflected the seasonal changes of water mass (Figure 6). Both genes were at least one order of magnitude more abundant in the FL fractions compared to the PA fractions. The dominant DMSP lyase gene in spring was *dddD* with an average of (2.08 ± 1.06) × 10^4^ copies ml^–1^ and (3.85 ± 3.57) × 10^3^ copies ml^–1^ in the FL (Figure 6a) and PA (Figure 6c) fractions, respectively. Lower *dddD* abundance (ca. 100–200 copies ml^–1^) was detected in January (winter) and June (summer) 2013. The analysis of the two samples collected from 50 m depth in June 2013 showed 2.18 × 10^3^ and 3.82 × 10^3^ copies ml^–1^ of the *dddD* gene. With the exception of the samples collected during spring, the *dddP* gene abundance exceeded the abundance of the *dddD* gene in both FL and PA fractions (1.94 × 10^2^–1.67 × 10^4^ copies ml^–1^ and 0–1.07 × 10^3^ copies ml^–1^, respectively).

In the case of DMSP demethylase, *dmdA* subclade D/1 was always the dominant *dmdA* subclade despite varying abundances in different seasons. The abundance of *dmdA* was at least one order of magnitude higher in the FL fractions (Figure 6b) than in the PA fractions (Figure 6d). In FL fractions, the abundance of *dmdA* subclade D/1 was lower in spring [average: (1.65 ± 0.78) × 10^4^ copies ml^–1^] than that in the other seasons [average: (5.55 ± 3.91) × 10^4^ copies ml^–1^]. Abundance of the *dmdA* subclade D/1 together with the *dmdA* subclade D/3 was high in early winter (December), especially in the offshore area (Station OT3) with maximum values of 1.62 × 10^5^ and 4.79 × 10^4^ copies ml^–1^, respectively. In contrast, *dmdA* subclade C/2 dominated in spring [average: (2.09 ± 0.52) × 10^4^ copies ml^–1^].

**FIGURE 7:**
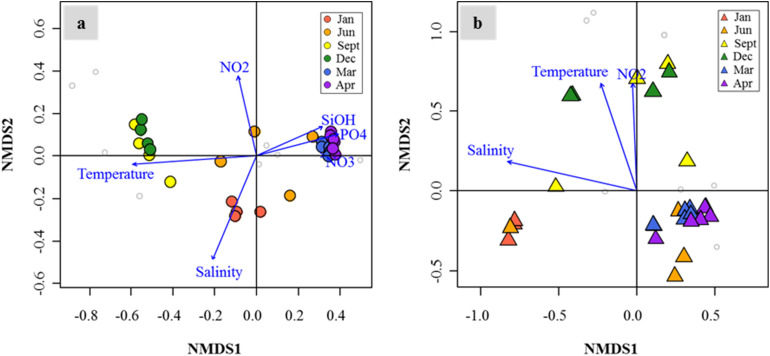
Nonmetric multidimensional scaling (NMDS) plot revealed temporal succession of dimethylsulfoniopropionate (DMSP) degradation gene distributions in the Sanriku Coast. After fitting with environmental parameters (*P* < 0.05), gene distributions in both free-living (FL) **(a)** and particle-associated (PA) **(b)** fractions were influenced significantly by temperature, salinity, and nitrite concentrations, while silicate, phosphate, and nitrate concentrations only influenced the FL fractions.

Distribution patterns of DMSP degradation genes in the FL and PA fractions and major drivers of environmental factors (*P* < 0.05) were analyzed by NMDS (Figures 7a,b). In both size fractions, the succession patterns of DMSP degradation genes showed high seasonality. Notably, the spring samples showed high similarities in both size fractions. Temperature, salinity, and nutrient concentrations (nitrate, nitrite, phosphate, and silicate concentrations) were identified as the major environmental drivers in the FL fractions. Similarly, in the PA fractions, the distribution of DMSP degradation genes was influenced by temperature, salinity, and nitrite concentration.

#### Correlation-Based Association Networks of Dimethylsulfoniopropionate Degradation Genes

**FIGURE 8:**
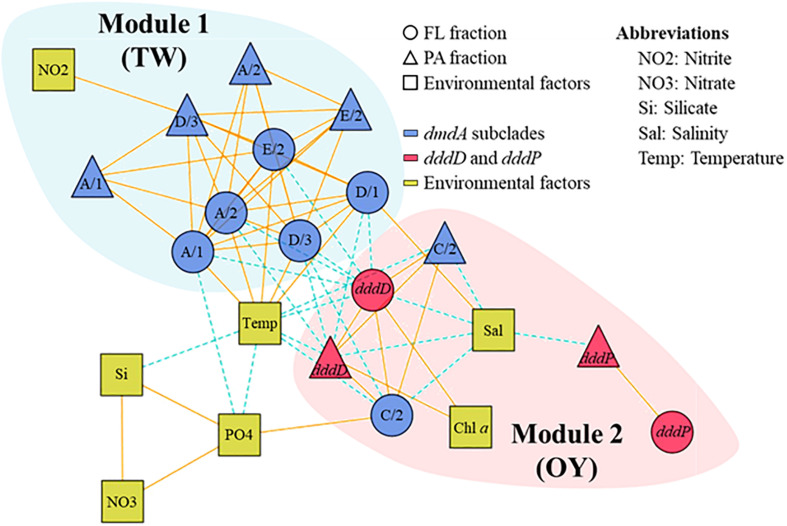
Association network of dimethylsulfoniopropionate (DMSP) degradation gene abundances in relation to environmental factors in the field study. The different modules are shaded with different colors. Most of the *dmdA* subclades were clustered in one module, while *dddD*, *dddP*, and *dmdA* subclade C/2 to a different module. Solid lines indicate positive correlations, while dashed lines indicate negative correlations.

To understand ecological interactions among DMSP degradation genes in relation to environmental conditions, we performed a correlation-based associated network analysis using the data regarding gene abundances and environmental factors (Figure 8). After filtering (*P* < 0.001 and *|ρ|* ≥ 0.6), a total of 70 correlations (links) among DMSP degradation genes and environmental factors (nodes) were found statistically significant. In the functional gene network, the DMSP degradation genes were divided into two different modules. DMSP demethylase genes, except the *dmdA* subclade C/2, were clustered into Module 1, while DMSP lyase genes and *dmdA* subclade C/2 were clustered into Module 2. Genes in Module 1 were more abundant in the samples when the sampling area was strongly influenced by TW Current, whereas genes in Module 2 were more abundant in the OY Current. Almost all correlations between members in Module 1 and Module 2 were negative. Subclades of *dmdA* within Module 1 were positively linked with each other. Most of the genes in Module 2, for instance *dddD*, *dmdA* subclade C/2, were negatively correlated with salinity. Temperature was positively correlated with *dmdA* subclade A/1, A/2, D/1, D/3, and E/2 while negatively correlated with *dddD* and *dmdA* subclade C/2. Abundance of *dddD* was positively linked with Chl *a* concentration in both fractions. Abundance of *dddP* was not linked to either *dmdA* or *dddD*. The association network showed higher values of modularity (0.29), clustering coefficient (0.62), and average path length (2.16) than the corresponding Erdös–Rényi random network (0.22, 0.30, and 1.77, respectively).

#### Variations of *dddD* in Oyashio Current Waters in Free-Living and Particle-Associated Fractions

The spring samples (March and April 2013) showed high abundance of *dddD* genes, thus, the *dddD* genes of the spring samples were subjected to deep-sequencing to compare the data with those from the mesocosm study. The similarities between the operational taxonomic units (OTUs) (97% similarity) obtained in this study and the closely related reference *dddD* gene sequences ranged from 60 to 80%. Meanwhile, the similarities between amino acid sequences translated from the OTUs and the closely related reference sequences ranged from 32 to 98%. The top 50 most abundant OTUs were chosen and calculated from summed relative abundance of all samples. Additionally, only OTUs which had a summed relative abundance 0.1% were chosen. Therefore, the relative abundance of individual OTU for each sample differed. Out of the top 50 most abundant gene clusters, only one OTU, representing less than 0.5% in total *dddD* reads, was assigned to Clade A (Supplementary Figure S3). Most of the major OTUs were assigned to Clade C and constituted more than 95% of the total *dddD* reads in each sample. The major OTUs also showed higher similarities to the sequences obtained from our mesocosm study than to the sequences obtained from previous studies (e.g., *Gammaproteobacterium* HTCC2207). Some major OTUs, for instance, OTU1, OTU2, and OTU4, appeared in all field samples and accounted for approximately 40–80% of total sequences. However, some OTUs (e.g., OTU20, OTU26, etc.) were only observed at certain sampling times. The NMDS plot of *dddD* genes compositions revealed differences between the PA and FL fractions in March and similarities between them in April (Supplementary Figure S4).

## Discussion

In this study, two different phytoplankton blooms were induced in mesocosm experiments, which enabled us to test the dynamics of DMSP-degrading bacteria in response to different phytoplankton regimes. In Mesocosm 1, a seed population was added to seawater after filtering other phytoplankton cells out with the assumption that the bloom would be dominated only by haptophytes. However, it was dominated not only by haptophytes (34% of total Chl *a*) but also diatoms (47–49%). On the other hand, the bloom of Mesocosm 2 was dominated by diatoms (45–49%) and green algae (35–38%), possibly prasinophytes and chlorophytes. In results, the two blooms consisted of mainly diatoms mixed with either haptophytes or green algae. It is noteworthy that the duplicate tanks of each Mesocosm presented quite consistent pigment compositions as well as Chl *a*, DMSP, and DMS fluctuation patterns. However, there were identified significant different patterns in the biochemical factors between the two mesocosm groups. For instance, DMSPp concentrations increased up to 3,000 nM in Mesocosm 2, which were approximately three times higher than the highest DMSPp concentration in Mesocosm 1. These differences were most likely caused by different phytoplankton communities between the two mesocosm groups, as DMSP production has been reported to vary between phytoplankton taxa (Matrai and Keller, 1994; Niki et al., 2000; Kasamatsu et al., 2004).

It has been suggested that variability in the DMSP supply could cause the changes in the abundance of *dddD-* and *dmdA-*possessing bacteria. The initial addition of seed coccolithophore caused approximately 20 times higher DMSPd concentration in Mesocosm 1 compared with that in Mesocosm 2 (Figures 2g,h). An effective bacterial DMSP cleavage, in accordance with the sharply increased DMS concentration, was observed at Day 2 in Mesocosm 1, while DMS concentrations did not increase significantly in Mesocosm 2 (Figures 2c,d). Since *Gephyrocapsa oceanica* NIES-353, the coccolithophore used in this study, could only produce negligible amounts of DMS (Niki et al., 2000); DMS could be mainly produced by bacterial DMSP cleavage. In agreement with the patterns of DMS concentrations, the abundance of *dddD* genes was also approximately three times higher in Mesocosm 1 than that in Mesocosm 2 (Figures 3a,d), indicating that DMS was mainly produced by the bacteria possessing *dddD* genes in this study.

DMSP demethylase was the most abundant DMSP degradation gene in both mesocosm and field studies, which is consistent with previous findings from open ocean and coastal waters (Howard et al., 2008; Varaljay et al., 2010, 2012; Levine et al., 2012). In the field study, the *dmdA* subclade D was always dominant, whereas the *dmdA* subclade C/2 became dominant in spring. The *dmdA* subclades D and C/2 could be possessed by different genotypes of SAR11 bacteria (Cui et al., 2015). Therefore, the bacterial community variations due to both seasonality and water current-derived environmental factors (Tada et al., 2011; Li et al., 2018) could result in variations of the *dmdA* subclades in our study area. Since SAR11 bacteria possess *dmdA* (Varaljay et al., 2010; Cui et al., 2015) and they are dependent on the reduced sulfur compounds (e.g., methionine, DMSP, etc.) for growth (Tripp et al., 2008; Sun et al., 2011), they could be assigned as the major DMSP degraders in both mesocosm and field studies. In contrast, the *dmdA* subclade A/1 and A/2 genes, which are believed to be carried by the marine Roseobacters (Varaljay et al., 2010), constituted a minimum proportion (<0.01% in mesocosm and < 4% in field) of the total DMSP degradation genes, despite marine Roseobacters being the dominant bacterial group. Therefore, it could be assumed that marine Roseobacters possessing *dmdA* genes play a less important role in degrading DMSP in this area.

We found an unexpectedly high representation of *dddD* genes in both the mesocosm experiment and the field study. Our results indicated *dddD* gene dominance and a significant correlation to DMS concentration in the mesocosm study, suggesting that bacteria possessing *dddD* genes were the major DMS producers in our study area. Of the different *ddd+* genes (*dddD*, *dddL*, *dddK*, *dddP*, *dddQ*, *dddY*, and *dddW*) (Moran et al., 2012; Sun et al., 2016), *dddP*, mainly found in the marine Roseobacters, has been reported to be the most frequently detected *ddd*+ gene in seawater (Todd et al., 2009; Peng et al., 2012; Cui et al., 2015). When the *dddP* gene was identified, it was reported to be the important “missing” gene coding for DMSP lyase because of its high abundance in the GOS metagenomic database (Howard et al., 2008). Subsequently, substantial studies have reported the abundance of *dddP* in seawater (Levine et al., 2012; Peng et al., 2012; Choi et al., 2015; Cui et al., 2015; Zeng et al., 2016). The *dddD* abundance was estimated to be only 1/10 of the abundance of the *dddP* gene in nearly all of the sampling sites in the Global Ocean Sampling (GOS), rendering it the least abundant *ddd*+ gene. Therefore, it has been commonly assumed that bacteria possessing *dddD* genes might play a minor role in DMSP cleavage (Howard et al., 2008; Todd et al., 2009). Only six *dddD* homologs were found in the GOS (Global Ocean Sampling Expedition) metagenomic database: three sequences from coastal environments, two sequences from embayment, and one sequence from mangrove (Howard et al., 2008; Todd et al., 2009). Several bacterial isolates, including *Marinomonas*, *Halomonas*, the marine Roseobacters, and *Pseudomonas*, that can grow well on DMSP as the sole carbon source, have been reported to contain *dddD* genes and can cleave DMSP to produce DMS (Curson et al., 2011). In contrast to the previous studies mentioned above which suggested that the abundance of *dddD* gene was extremely low and *dddP* gene was abundant in both coastal and the open oceans, our results showed that the abundance of *dddD* genes exceeded those of *dddP* in FL and PA fractions in our mesocosm study (Figure 3) and in the spring samples of the field study (Figure 6). In the mesocosm study, the total *dddD* abundance, combining the FL and PA fractions, was significantly positively correlated with the concentration of DMS. These results give prominence to *dddD* as a possible major DMSP lyase gene in certain seawater environments.

*dddD* genes can be divided into several clades: Clade A was mainly composed of marine Roseobacters, Clade B of Rhizobacteria, and Clade C of a wide variety of *Gammaproteobacteria*. The initial *dddD* genes in the mesocosm and field studies belonged to Clade C (Figure 4b and Supplementary Figure S3), indicating that *Gammaproteobacteria* possessing *dddD* play an essential role in DMS production in natural seawater. Culture-based lab studies have reported that some *Gammaproteobacteria*, for instance *Oceanimonas*, *Marinomonas*, and *Pseudomonas*, possessed *dddD* genes, and this gene played a key role in DMS production (Curson et al., 2011), indicating that *Gammaproteobacteria* has the potential to produce DMS *via dddD* genes. In the mesocosm study, Chl *a* concentration increased toward the end of the experiments (Figures 2a,b), and one of the dominant bacterial groups at Day 9 was the marine Roseobacters (or *Rhodobacteraceae*) (Supplementary Figure S5). This change toward the end of the mesocosm experiment corresponded to the change of a major *dddD* gene for Clade A, indicating that the marine Roseobacters possessing *dddD* can be an essential DMS producer in phytoplankton blooming conditions. The marine Roseobacters have been reported to form intimate relationships with other phytoplankton cells (Buchan et al., 2014) and are characterized by a high proliferation rate (Tada et al., 2011). Therefore, phytoplankton-derived DMSP could be more easily utilized by the nearby marine Roseobacters to produce DMS.

In Mesocosm 2, the increase in the abundance of *dddD* genes in the PA fractions corresponded to the increase of the DMS concentration in the latter half of the experiment, and Clade A was the dominant *dddD* genes in the PA fractions (Figure 4d). These results suggest that the marine Roseobacters associated with particles play a significant role in the DMS production. Large aggregates occurred from Day 5 (Supplementary Figure S2) and a drastic decrease in Chl *a* concentrations was observed in the middle and later phases of the experiment (Figures 2a,b). Therefore, a large amount of phytoplankton-derived organic matter, including DMSP, could have been released into the seawater, accelerating the proliferation of phytoplankton-associated bacteria. The bacterial community analysis based on the 16S rRNA gene in Mesocosm 2 revealed an increase of *Rhodobacteraceae* including *Roseobacter* bacteria (Supplementary Figure S5) in the latter phase of the experiment (FL, 36%; PA, 62% in total bacteria abundance). Therefore, high concentrations of DMSP produced by phytoplankton decomposition acted as a source of proliferation of the marine Roseobacters bacteria possessing *dddD* that subsequently produced a significant amount of DMS.

Interconnectivity within bacteria involved in DMSP metabolism is complicated and depends on environmental conditions. This is apparent on the effects that habitat filtering and niche partitioning have on DMSP degraders in relation to the variability in biological and physicochemical factors caused by seasonal changes of the water current systems. An association network visualized the interactions among bacteria possessing DMSP degradation genes in relation to environmental factors, which allowed us to identify the closely related DMSP degradation gene clusters. Similar to other association networks based on 16S rRNA genes in aquatic ecosystems (Chow et al., 2014; Hu et al., 2017), the network of DMSP degradation genes were divided into TW Current-related module (Module 1) and OY Current-related module (Module 2) (Figure 8). A module is a collection of nodes with a similar tendency in the ecological network. The components of each module were functionally related; that is, genes sharing similar functions (e.g., *dmdA* subclades) were clustered in the same module. These results indicated that functional relatedness determined their interconnectivity in natural environments, suggesting the importance of these gene functions in characterizing their host bacteria. Temperature and salinity positively correlated with most of the *dmdA* subclades but were negatively associated with *dddD* genes and the *dmdA* subclade C/2. These two factors are the key indices to distinguish among different water current systems (Hanawa and Mitsudera, 1986; Wagawa et al., 2015). The OY Current carried cold, low–salinity, and nutrient-rich water, while the TW Current carried warm, saline, and relatively low in nutrients water (Figure 5). The modules involved in DMSP metabolism changed in accordance to the changes in different nutrients and Chl *a* concentrations of the water currents. Multidimensional NMDS analysis also revealed that the physicochemical factors (temperature, salinity, and nutrient concentrations) were responsible for the succession of DMSP degradation genes (Figure 7).

Previous studies, including this study, explored the abundance of DMSP degradation genes based on primer sets which were designed to target the major DMSP degraders, for instance, SAR11 and Roseobacters. Therefore, it is possible that many other bacteria possessing DMSP degradation genes, which were not covered by current primer sets, could play essential roles in DMSP metabolism, and this remains as future work to uncover.

## Conclusion

In conclusion, our study uncovered that the distribution and abundance of *dmdA*, *dddD*, and *dddP* genes largely varied depending on the changes of biological and physicochemical factors in the seawater environment but shared high interconnectivities between functionally related genes. Variability in DMSP supply due to the change of phytoplankton species and their blooming conditions changed the dynamics of DMSP-degrading bacteria, which led to the change of DMS concentration in seawater. Also, the change of environmental parameters due to the shift of water mass changed their dynamics as well, implying the effect of habitat filtering and niche partitioning. More specifically, we firstly found that the abundance of *dddD* genes showed a positive correlation with the DMS concentration, indicating that bacteria possessing *dddD* genes can be a significant DMS producer in the natural seawater. Our results also revealed possible bacterial taxa responsible for DMSP consumption and DMS production in relation to the change of environmental factors. SAR11 bacteria possessing the *dmdA* subclade C/2 are possibly the major DMSP consumer and gammaproteobacteria possessing *dddD* the major DMS producer in the OY Current characterized by cold, low–salinity, and nutrient-rich water. On the other hand, possibly SAR11 bacteria possessing *dmdA* subclade D are the major DMSP consumer and the marine Roseobacters possessing *dddP* are the major DMS producer in the TW Current characterized by warm, saline, and relatively nutrient-poor water. Also, the marine Roseobacters possessing *dddD* can mainly contribute to the DMS production under phytoplankton blooming conditions by associating with phytoplankton cells and marine particles. Future studies are needed to explore the gene expression levels of *dmdA*, *dddD*, and *dddP* genes and to identify the hidden mechanisms of DMSP degradation and DMSP production in the Sanriku Coast.

## Data Availability Statement

The datasets generated for this study can be found in the NCBI under project number PRJNA547684.

## Author Contributions

YC and KH designed the study and led to write the manuscript. YC and S-KW made substantial contributions to gene analysis. YC, S-JC, H-GL, C-YA, and H-MO have made contribution to the network analysis. RK, HF, TN, and KK have made contribution to the field study and measurements of environmental variables. AM, YT, IN, YS-T, KS, and KH have made contribution to the mesocosm study and measurements of DMS, phytoplankton pigments and other parameters. All authors listed have made a substantial, direct and intellectual contribution to the work, and approved it for publication.

## Conflict of Interest

The authors declare that the research was conducted in the absence of any commercial or financial relationships that could be construed as a potential conflict of interest.
