# Supplementary material for: Distribution of Dimethylsulfoniopropionate Degradation Genes Reflects Strong Water Current Dependencies in the Sanriku Coastal Region in Japan: From Mesocosm to Field Study
Source: Front Microbiol. 2020 Jul 13;11:1372. doi: 10.3389/fmicb.2020.01372 (PMC7370799; doi:10.3389/fmicb.2020.01372)
Supplement: Supplementary file 1 [file Data_Sheet_1.docx]

Distribution of dimethylsulfoniopropionate degradation genes reflects strong water current dependencies in the Sanriku coastal region in Japan: from mesocosm to field study

Yingshun Cui^1†*^, Shu-Kuan Wong^1^, Ryo Kaneko^1,2^, Ayako Mouri^1^, Yuya Tada^1,3^, Ippei Nagao^4^, Seong-Jun Chun^5,6^, Hyung-Gwan Lee^5^, Chi-Yong Ahn^5^, Hee-Mock Oh^5^, Yuki Sato-Takabe^7^, Koji Suzuki^7^, Hideki Fukuda^8^, Toshi Nagata^9^, Kazuhiro Kogure^1^ and Koji Hamasaki^1,10*^

^1^Marine Microbiology, Department of Marine Ecosystem Dynamics, Atmosphere and Ocean Research Institute, The University of Tokyo, Kashiwa, Japan. ^2^National Institute of Polar Research, Tachikawa, Japan. ^3^National Institute for Minamata Disease, Kumamoto, Japan. ^4^Graduate School of Environmental Studies, Nagoya University, Japan. ^5^Cell Factory Research Center, Korea Research Institute of Bioscience and Biotechnology (KRIBB), Daejeon, South Korea. ^6^National Institute of Ecology, Seocheon-gun, South Korea. ^7^Faculty of Environmental Earth Science, Hokkaido University, Sapporo, Japan. ^8^International Coastal Research Center, Atmosphere and Ocean Research Institute, The University of Tokyo, Kashiwa, Japan. ^9^Marine Biogeochemistry, Department of Chemical Oceanography, Atmosphere and Ocean Research Institute, The University of Tokyo, Kashiwa, Japan. ^10^Collaborative Research Institute for Innovative Microbiology, The University of Tokyo, Tokyo, Japan.

***Correspondence**:

Yingshun Cui

Marine Microbiology

Department of Marine Ecosystem Dynamics

Atmosphere and Ocean Research Institute, University of Tokyo

5-1-5, Kashiwanoha, Kashiwa-shi, Chiba 277-8564 Japan

E-mail : yscui415@kribb.re.kr

Koji Hamasaki

Marine Microbiology

Department of Marine Ecosystem Dynamics

Atmosphere and Ocean Research Institute, The University of Tokyo

5-1-5, Kashiwanoha, Kashiwa-shi, Chiba 277-8564 Japan

E-mail: [hamasaki@aori.u-tokyo.ac.jp](mailto:hamasaki@aori.u-tokyo.ac.jp)

^†^Cell Factory Research Center, Korea Research Institute of Bioscience and Biotechnology (KRIBB), 125 Gwahak-ro, Yuseong-gu, Daejeon 34141, South Korea

**Supplementary methods**

**Bacterial 16S rRNA gene analysis**

Pyrosequencing was performed as described previously (Cui *et al*., 2015). Briefly, bacterial 16S rRNA genes were amplified using the barcoded primer set 27F and 519R (targeting the V1-V3 regions) (Lane, 1991;Kim *et al*., 2011) for 20 cycles. PCR products were prepared in triplicates and pooled for purification. The pooled PCR products were purified using the Ampure system (Agencourt Bioscience Corporation). The prepared DNA libraries were sequenced using the 454 GS Junior technology (Roche Applied Science, Indianapolis, IN) according to the manufacturer’s instructions.

Bacterial 16S rRNA gene sequences were processed using mother v. 1.39.3 (Schloss *et al*., 2009) following the standard operating procedure (SOP) proposed by Schloss *et al*. (Schloss *et al*., 2011). Sequences were removed from the analysis if they had a read quality score under 30, contained ambiguous characters, contained more than two mismatches to the forward primer or one mismatch to the barcode, or were under 250 bp or over 550 bp. The optimized minimum read length was 266 bp after barcode and primer sequences were trimmed. Greengene database (gg-13-5-99) was used to align and classify the sequences. The similarity cut-off of >97% was used to assign the sequences into OTUs. Obtained bacterial 16S rRNA sequences and accompanying metadata have been deposited in the Sequence Read Archive (SRA) of NCBI under project number PRJNA547684.

**Gene analysis of the *dddD* sequences obtained from pyrosequencing**

The obtained *dddD* gene sequences were processed using mother v. 1.39.3 (Schloss *et al*., 2009). Clone library sequences (mesocosm study) and the *dddD* reference sequences obtained from NCBI were aligned using CLUSTAL X and used as a database to analyse the *dddD* sequences obtained in pyrosequencing. A taxonomy file containing the taxonomic sequence affiliations for the sequences was constructed from the *dddD* database. The aligned sequence database along with the taxonomy file were imported into mothur v. 1.39.3 (Schloss *et al*., 2009) for subsequent sequence analysis of the *dddD* genes from our samples according to the standard operating procedure (SOP) proposed by Schloss *et al*. (Schloss *et al*., 2011). The sequences were aligned with Needleman-Wunsch pairwise alignment method with +1, -1, and -2 for matches, mismatches, and gaps, respectively. We used k-mer searching with 8-mers for alignment.

**Supplementary table and figures**

**Table S1. Diagnostic pigments of phytoplankton and their taxonomic association in the mesocosm study at Day 7.**

| **Diagnositic pigment** | **Taxonomic association** | **Mesocosm 1** | | **Mesocosm 2** | |
| --- | --- | --- | --- | --- | --- |
|  |  | Tank A [μg l^-1^] | Tank B [μg l^-1^] | Tank C [μg l^-1^] | Tank D [μg l^-1^] |
| Chl.*b* | Green algae | 1.18 | 1.64 | 1.80 | 1.59 |
| 19’-Hexanoyloxyfucoxanthin | Haptophytes | 2.34 | 3.07 | 1.04 | 0.56 |
| Fucoxanthin | Diatoms | 3.40 | 4.18 | 2.30 | 2.05 |

**Figure S1. Sampling site of the field study. The mesocosm study was performed near the OT3 sites.**


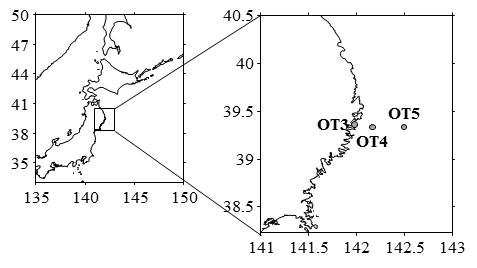


**Figure S2. Bacterial abundance in FL and PA fractions in the two mesocosm systems: a) Tank A; b) Tank C; c) Tank C; d) Tank D.**


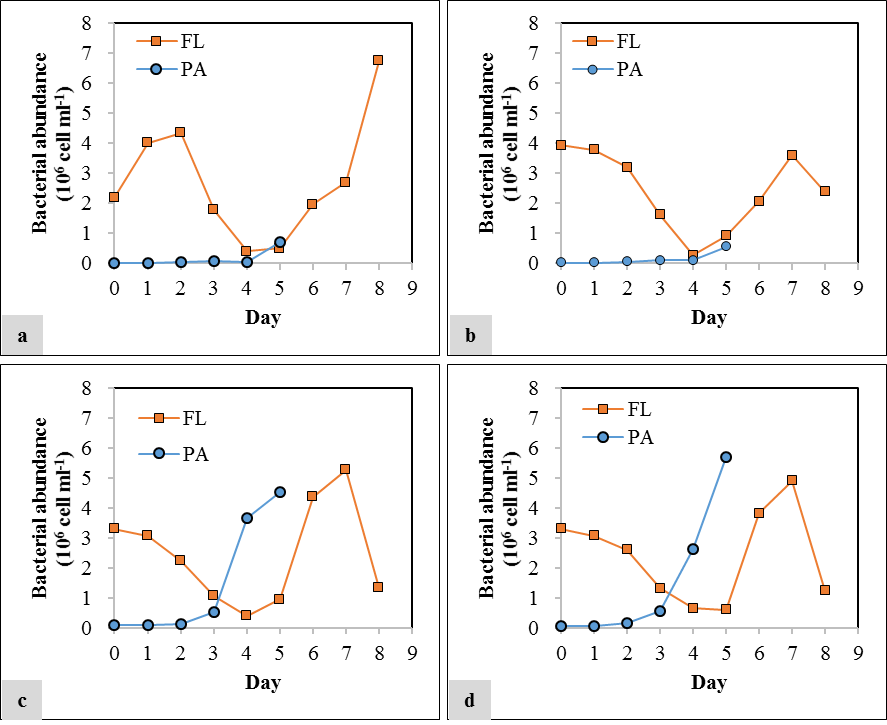


**Figure S3. Neighbor-joining phylogenetic tree of the *dddD* gene OTUs (red) and *dddD* clusters (blue) obtained from the mesocosm experiment with reference sequences obtained from NCBI gene bank. Clones with ≥ 97% sequence similarity were assigned to the same cluster. Numbers in parentheses next to each cluster indicate the number of sequences in that cluster. Bootstrap values (expressed as percentages of 1000 replications) >50% are shown at branch points. Each clade is shaded accordingly with different colours.**


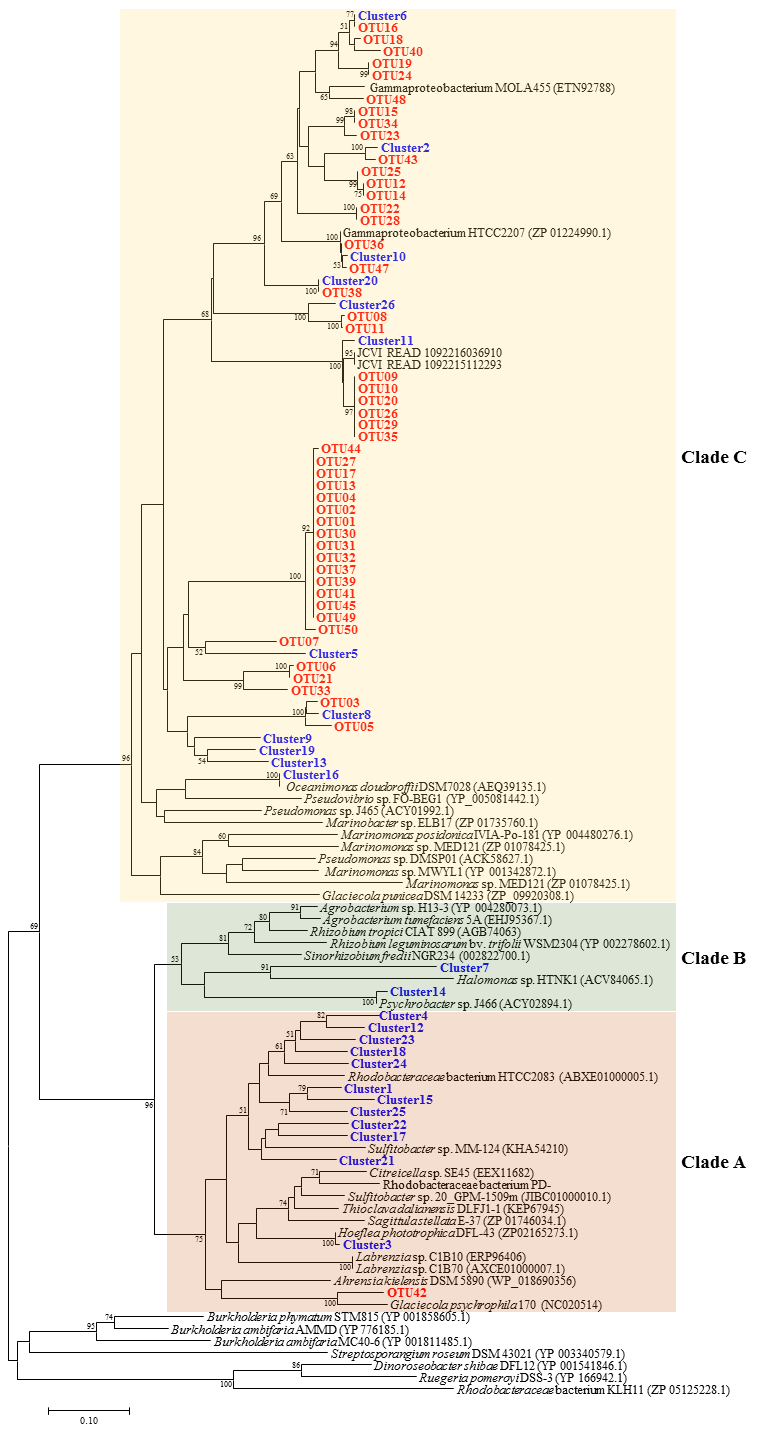


**Figure S4. NMDS plot revealed differences in the *dddD* genotypes from the FL and PA fractions in March and April 2013.**


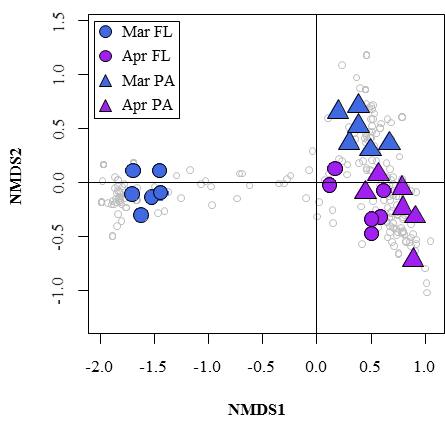


**Figure S5. Bacterial community compositions (family level) in FL (a) and PA (b) fractions at Day 0, 2, and 9. M1, Mesocosm 1; M2, Mesocosm 2.**


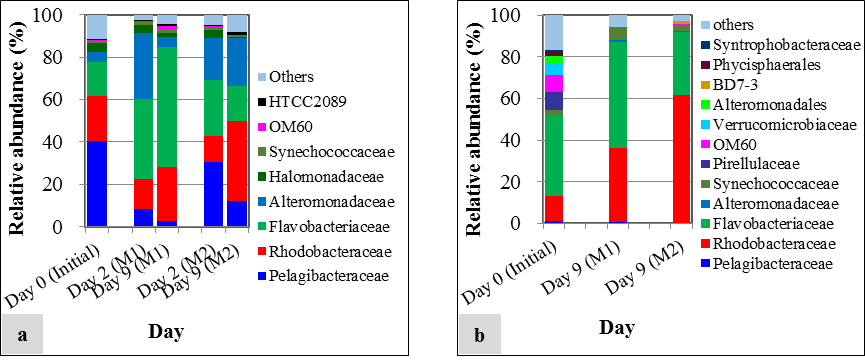


Reference

Cui, Y., Suzuki, S., Omori, Y., Wong, S.K., Ijichi, M., Kaneko, R., Kameyama, S., Tanimoto, H., and Hamasaki, K. (2015). Abundance and distribution of dimethylsulfoniopropionate degradation genes and the corresponding bacterial community structure at dimethyl sulfide hot spots in the tropical and subtropical pacific ocean. *Appl Environ Microbiol* 81**,** 4184-4194.

Kim, M., Morrison, M., and Yu, Z.T. (2011). Evaluation of different partial 16S rRNA gene sequence regions for phylogenetic analysis of microbiomes. *J Microbiol Meth* 84**,** 81-87.

Lane, D. (1991). 16S/23S rRNA sequencing. Nucleic acid techniques in bacterial systematics In: Stackebrandt, E. and Goodfellow, M., Eds., Nucleic acid techniques in bacterial systematic, John Wiley and Sons, New York, 115-175.

Schloss, P.D., Gevers, D., and Westcott, S.L. (2011). Reducing the Effects of PCR Amplification and Sequencing Artifacts on 16S rRNA-Based Studies. *Plos One* 6**,** e27310.

Schloss, P.D., Westcott, S.L., Ryabin, T., Hall, J.R., Hartmann, M., Hollister, E.B., Lesniewski, R.A., Oakley, B.B., Parks, D.H., Robinson, C.J., Sahl, J.W., Stres, B., Thallinger, G.G., Van Horn, D.J., and Weber, C.F. (2009). Introducing mothur: open-source, platform-independent, community-supported software for describing and comparing microbial communities. *Appl Environ Microbiol* 75**,** 7537-7541.
